# Quantitative analysis on electric dipole energy in Rashba band splitting

**DOI:** 10.1038/srep13488

**Published:** 2015-09-01

**Authors:** Jisook Hong, Jun-Won Rhim, Changyoung Kim, Seung Ryong Park, Ji Hoon Shim

**Affiliations:** 1 Department of Chemistry, Pohang University of Science and Technology, Pohang 790-784, Korea; 2 School of Physics, Korea Institute for Advanced Study, Seoul 130-722, Korea; 3 Institute of Physics and Applied Physics, Yonsei University, Seoul 120-749, Korea; 4 Department of Physics, Incheon National University, Incheon 406-772, Korea; 5 Department of Physics and Division of Advanced Nuclear Engineering, Pohang University of Science and Technology, Pohang 790-784, Korea

## Abstract

We report on quantitative comparison between the electric dipole energy and the Rashba band splitting in model systems of Bi and Sb triangular monolayers under a perpendicular electric field. We used both first-principles and tight binding calculations on p-orbitals with spin-orbit coupling. First-principles calculation shows Rashba band splitting in both systems. It also shows asymmetric charge distributions in the Rashba split bands which are induced by the orbital angular momentum. We calculated the electric dipole energies from coupling of the asymmetric charge distribution and external electric field, and compared it to the Rashba splitting. Remarkably, the total split energy is found to come mostly from the difference in the electric dipole energy for both Bi and Sb systems. A perturbative approach for long wave length limit starting from tight binding calculation also supports that the Rashba band splitting originates mostly from the electric dipole energy difference in the strong atomic spin-orbit coupling regime.

Rashba band splitting in a system with an inversion symmetry breaking (ISB) such as material surfaces and hetero-structures recently has drawn much attention in condensed matter physics community^1^,^2^,^3^,^4^,^5^. In addition to its fundamental importance, it is believed that it plays a vital role in spin-orbit torque in spintronic materials^3^. It is thus important to thoroughly understand the origin of the Rashba band splitting. Tight binding and first-principles calculations reproduce very well the experimentally observed Rashba band splittings on material surfaces^6^,^7^,^8^. Especially, tight binding calculations show that atomic spin-orbit coupling (SOC) is a crucial parameter in the Rashba band splitting^6^. However, it had been unclear which interaction among many is crucial for the Rashba band splitting.

It was recently found that the atomic orbital angular momentum (OAM) exists in the presence of the ISB and that it induces an asymmetric charge distribution for non-zero crystal momentum^9^. The dipole energy from the interaction between asymmetric charge distribution and the electric field from the ISB is proposed to be responsible for the Rashba band splitting in the strong SOC case. The proposed effective Hamiltonian for the Rashba band splitting is *H* = *α***L** · **S** − *α*_k_(**k** **×** **L**) · **E**_s_, where *α*, **L**, **S**, α_K_, **k**, and **E**_s_ are SOC parameter, OAM, spin angular momentum (SAM), coupling parameter for electric dipole interaction, crystal momentum and electric field induced by the ISB, respectively^10^,^11^. The first term in the Hamiltonian is the usual atomic SOC while the second term represents the newly proposed electric dipole energy. It has been previously conjectured that the atomic SOC determines the Rashba band splitting in weak atomic SOC regime while the new dipole energy term does so in strong atomic SOC regime^11^,^12^. In both cases, the existence of the OAM is essential.

The existence of the OAM in Rashba bands was shown by tight binding and first-principles calculations^9^,^13^. It was also experimentally supported in the observation of strong circular dichroism in angle-resolved photoemission (ARPES)^10^,^11^,^14^,^15^. While the existence of the OAM is certain, its role in the Rashba band splitting does not appear to be fully accepted. Part of the reason could be from the fact that quantitative estimation of the OAM contribution based on, for example, first-principles calculation has not been performed. Therefore, a quantitative comparison between the electric dipole energy difference and the Rashba band splitting in neighboring bands is desired, especially in the strong atomic SOC case. Significance in the electric dipole energy difference compared to the total Rashba band splitting energy should prove the validity of the OAM based effective Rashba model.

To show the importance of the electric dipole energy in a quantitative way, we performed first-principles and tight binding calculations on Bi and Sb monolayers with an external field. Use of monolayers with an external field is to mimic the surface state without dealing with complicated bulk states. This is a simple enough model for the purpose of our research to explore the origin of the Rashba band splitting of the surface states. Both first-principles and tight binding calculations are complementary to each other. The electric dipole energy is estimated by using the wave functions from first-principles calculation, which is not model-dependent, while tight binding calculations allow us to have more intuitive analysis on the band structures and wave functions. The outcome from the interaction between the asymmetric charge distribution and the electric field is responsible for the Rashba band splitting in Bi and Sb monolayers.

## Results and Discussions

### First-principle calculations

**Figure 1:**
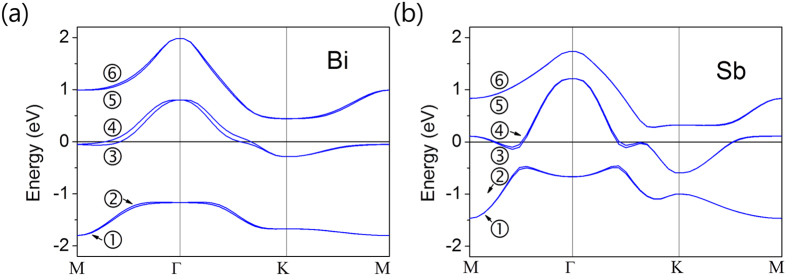
Calculated band structures. Band structure of (**a**) Bi single layer and (**b**) Sb single layer under the electric field 0.5 V/Å along the perpendicular direction to the layer. Bands 3 and 4 show the largest splitting and the others show small splitting.

We present in Fig. 1 the band structures of Bi and Sb single layers under an electric field 0.5 V/Å along the direction perpendicular to the layers. The six bands are composed of *p*-orbitals of Bi or Sb atoms. Bands 1 and 2 are mainly of *J* ≈ 1/2 character while bands 3 to 6 come from mainly *J* ≈ 3/2 states. We can see the Rashba splitting in the band structure near the Γ and M points which are time reversal invariant momenta (TRIM) of the triangular lattice. Bi single layer whose atomic SOC is stronger than that of Sb shows a larger Rashba splitting in its band structure, which indicates that the magnitude of the Rashba splitting is clearly related to the atomic SOC strength. Indeed, the Rashba splitting observed at Cu, Ag, and Au surfaces also show such correlation between the atomic SOC and the size of the Rashba splitting^16^.

**Figure 2:**
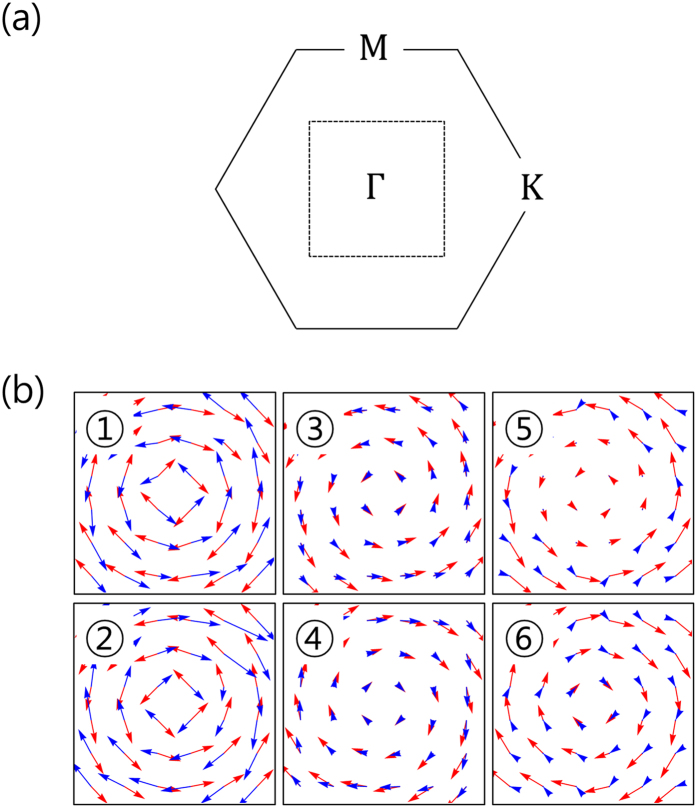
SAM and OAM of Bi triangular lattice under the field. SAM and OAM of bands 1 to 6 of Bi triangular lattice near the Γ point are shown in (**b**). The numbers indicate the band indices shown in Fig. 1. Red arrows are SAM and blue arrows are OAM. The 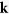 space region for (**b**) is shown in (**a**) as dotted square.

In Fig. 2, we plot the expectation values of in-plane components of SAM and OAM of the Bi single layer near the Γ point. The SAM and OAM for Sb single layer (not shown here) have the same trends as those of Bi single layer. The only difference is the smaller OAM magnitude for Sb compared with Bi, which might be the result of the small SOC in Sb. All the OAM patterns of the bands show chiral structures around the Γ point and the chiral directions of adjacent bands are opposite to each other. SAM also has similar patterns to those of OAM because of the strong SOC. SAM for *J* ≈ 1/2 bands (bands 1 and 2) are antiparallel to OAM while they are almost parallel to the OAM directions in *J* ≈ 3/2 bands (bands 3 to 6).

We notice that while the OAM magnitude is the largest in the bands 1 and 2, the largest band splitting exists in bands 3 and 4. Therefore, the magnitude of OAM cannot be directly linked to the size of the Rashba band splitting. Instead, asymmetric charge distribution, which results from interference of adjacent atomic orbitals with OAM, is found to be proportional to the size of the Rashba band splitting. Therefore, the overlap between adjacent atomic orbitals is also an important factor in the determination of the asymmetric charge distribution, hence the Rashba band splitting. We believe that the electric dipole moments of the states in bands 3 and 4 become stronger than those in bands 1 and 2 due to larger overlap between adjacent atomic orbitals. We will discuss this issue in more detail below.

**Figure 3:**
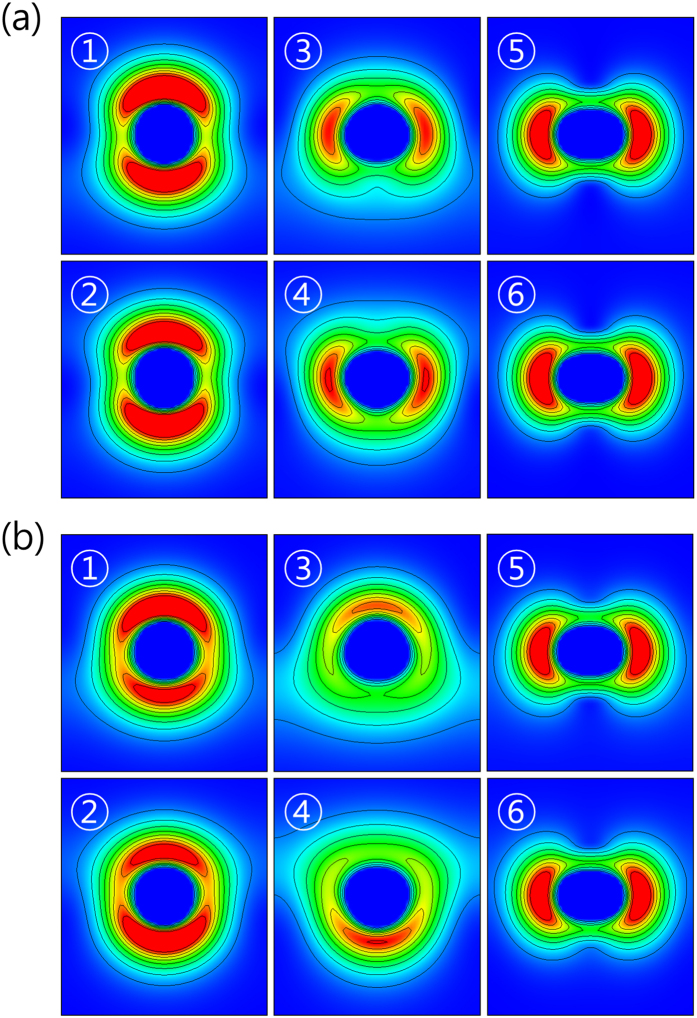
Charge density around the Bi atom. Charge density around the Bi atom in xz-plane when the crystal momentum **k** is apart from Γ point as much as (**a**) 0.2π/a and (**b**) 0.4π/a directed at M point. The numbers indicate the band indices shown in Fig. 1. Red color means dense charge density and blue color is rare charge density.

We plot the charge densities around a Bi atom for crystal momenta *k* = 0.2π/a and 0.4π/a along the Γ-M direction in Fig. 3. Bands 3 and 4 show clear charge asymmetry along the *z-*direction. Other bands show rather small asymmetry. So, it can be noticed that the magnitude of band splitting is closely related to the electric dipole moment of a state in the band. Bands 1 and 2 have a shape of *p*_*z*_ orbital and therefore small orbital overlap between nearest atoms, resulting in small charge asymmetry, which reveals the importance of the orbital overlap for the formation of the electric dipole. Because of this small electric dipole moment, bands 1 and 2 have small Rashba splitting. Bands 3 and 4 have more charge along the in-plane direction (more overlap between neighboring orbitals) as well as relatively large in-plane components of the OAM. Therefore they have much larger charge asymmetry along the *z-*direction and band splitting. The top bands (5 and 6) have small charge asymmetry due to the smallest in-plane component of the OAM. When the crystal momentum *k* becomes twice larger (*k* = 0.4π/a in Fig. 3 (b)), the charge asymmetry becomes more significant. This result is consistent with the Rashba splitting being proportional to the *k* value as shown in Fig. 1. The same trend is observed in Sb single layer. The only difference is that the size of the dipole moment and band splitting is smaller because of the smaller SOC.

**Figure 4:**
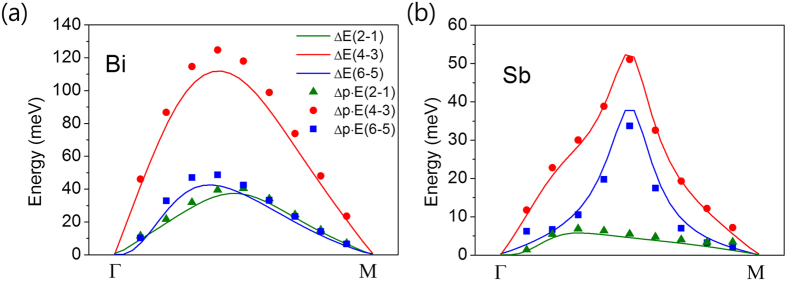
Comparisons between the band splitting and dipole energy. Comparisons between the band splitting (solid lines) and dipole energy difference (dots) in (**a**) Bi and (**b**) Sb systems as varying the crystal momentum **k** from Γ to M.

To ensure the origin of Rashba band splitting, we compare electric dipole energy difference (dots) Δ**P** · **E**_ext_ with the Rashba band splitting (solid lines) for both Bi and Sb layers as varying the crystal momentum **k** from Γ to M point as shown in Fig. 4. Overall, the electric dipole energy difference and the Rashba band splitting are well consistent in all range of crystal momentum for both systems. Small discrepancy can be attributed into two factors. One is the atomic SOC, α**L** · **S**. The other is the electric-field screening effect due to charge redistribution in Bi or Sb layers by the external electric field. Mostly, the electric dipole energy difference is larger than the Rashba band splitting in Bi layer. It is mainly because of the screening effect. We confirm that the Rashba band splitting mostly originates from the electric dipole energy difference in both Bi and Sb layers, which are considered as in the strong SOC regime. In the next section, we investigated more on the role of the dipole moment in the Rashba splitting and its correlation to SOC by using tight binding approach.

### Tight Binding Analysis

#### Perturbation theory

We consider a tight binding model for the triangular lattice which consists of Bi or Sb atoms. The electronic properties of them are well-described by *p*-orbitals while other ones reside in energy levels much far from the Fermi level^17^. The tight binding Hamiltonian is given by


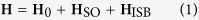


where **H**_0_ is the hopping processes between *p*-orbitals on the two dimensional triangular lattice without any external fields, 
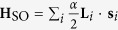
 is the atomic spin-orbit interaction and **H**_ISB_ is the ISB term coming from external effects such as electric field or the substrate.

**Figure 5:**
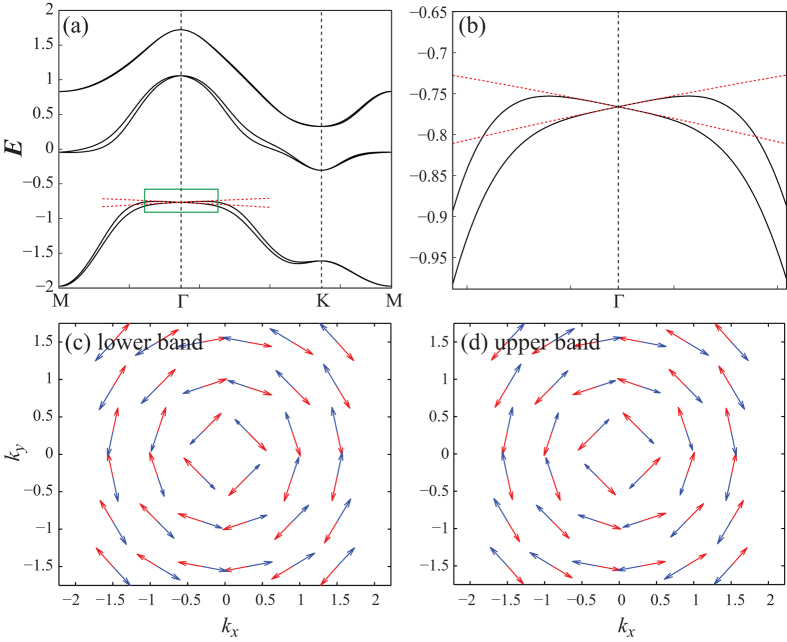
Electronic structures from tight binding approximation. Band structures for a set of tight binding parameters 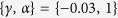 in the unit of eV. The regions in the green boxes in (**a**) is highlighted in (**b**). The red dashed bands are obtained from the perturbation theory. For two lowest bands, we plot their OAM (blue) and SAM (red) structures in (**c**) and (**d**) each.

The bare Hamiltonian **H**_0_ reads


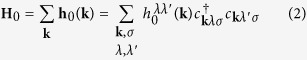


where *λ* = *x*, *y*, *z* is the orbital index and *σ* = ↑,↓ is the spin index. The matrix elements 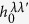 are given by


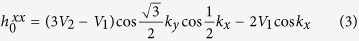



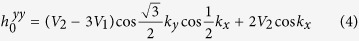



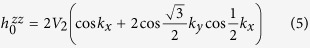



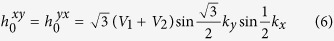


whose long wavelength limits are expressed by 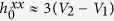  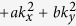,  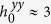 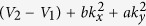,  
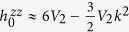
  and  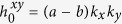.Here, *a* *=* 3(3*V*_1_−*V*_2_)/8, *b* *=* 3(*V*_1_−3*V*_2_)/8 and 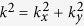. Other elements (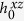 and 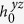) are vanishing because the hopping between *p*_*x*_ or *p*_*y*_ and *p*_*z*_ is forbidden by the symmetry. *V*_1_ and *V*_2_ represent the *σ* and *π* bonding between *p* orbitals. A set of binding parameters, 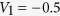 eV and 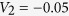 eV, yields band structures consistent with the DFT results as shown in Fig. 5. Accurate tight binding parameters are not pursued here since the main purpose of this section is to understand the relation between the dipole energy difference and the Rashba band splitting.

We suppose that the inversion symmetry with respect to the *xy*-plane is broken so that


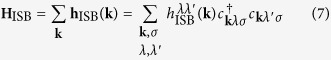


where


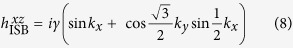



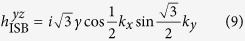


which satisfy 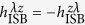 for *λ* ≠ *z*. Here *γ* measures the energy scale of the applied electric field. Near Γ point, they are approximated to 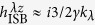. Other matrix elements (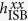, 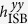, 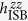, 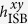 and 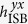) are vanishing.

At Γ point, one can obtain the analytic solution of **h**_0_(0) + **h**_SO_(0). As an example, we focus on the lowest two degenerate levels for given tight binding parameters above. The eigenenergy is


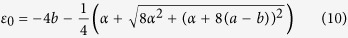


and corresponding eigenvectors are


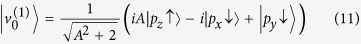



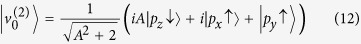


where


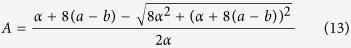


Then, near Γ point, the other terms *h*_0_(**k**)−*h*_0_(0) + *h*_ISB_(**k**) can be considered as a perturbation and we obtain effective 2×2 Hamiltonian for those two bands as


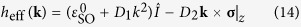


where 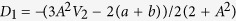, 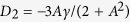 and 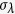 is the Pauli matrix. The eigenvalues are


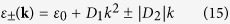


and corresponding eigenvectors are


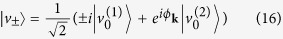


where 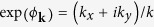. With these, one can obtain the OAM of each band as follows.


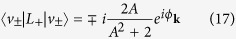


The directions of the OAM of the two lowest bands are opposite to each other when this perturbation scheme is valid.

The results on the electronic structures from our tight binding approximation are presented in Fig. 5. One can note that the tight binding bands and their OAM structures show good agreement with the first-principles results in the previous section. Also, we check the analytic results in the long wavelength limit works very well as indicated by red dotted lines in Fig. 5.

#### Dipole energy

In this section, we prove that the band splitting is closely related to the dipole energy difference. Here, we regard **h**_ISB_(**k**) as a perturbation to explain the splitting of two degenerate multi-orbital bands by the ISB. This is a more general consideration compared with the previous section and its results hold throughout whole Brillouin zone if *γ* is much smaller than the spin-orbit interaction.

Let us denote the Bloch states on two split bands as 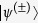 whose spatial representations are given by


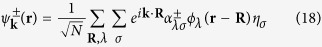


where *λ* *=* *x*, *y*, *z* is the orbital index, *ϕ*_*λ*_ is the real space wave function of *p*_λ_-orbital and *η*_*σ*_ is the spinor. Then the dipole energy 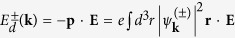 becomes


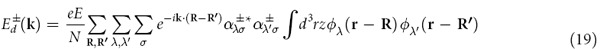


where the electric field is in *z* direction (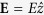). One of the important properties of the *p*-orbitals is that they cannot give a finite dipole moment if they are in the same site. As a result, the integral of the above is vanishing if **R** = **R**′. If we consider the overlaps of wave functions between the nearest neighboring sites as leading contributions,


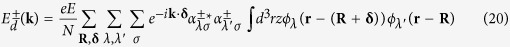



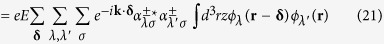


where **δ** represents the six nearest neighboring sites of each lattice point. In the last integral, one of *λ* and *λ*′ (not both) should be *z* so that the integral is an even function in *z* direction. Then,


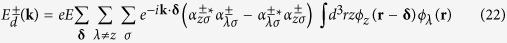


where we have used the fact that *ϕ*_*λ*_ is an odd function in *λ* direction. One can note that, when 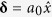, the integral 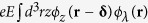 is just the hopping parameter 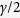 for the ISB which measures the matrix element of the applied electric potential. If we exploit the vectorial property of *p* orbitals, the dipole energy becomes


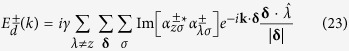



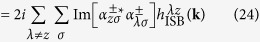


Then, the dipole energy difference, 
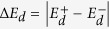
, at each momentum **k** is


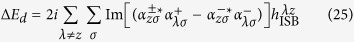


Near Γ point (*k*  *α*/*γ*), the eigenvectors for the split bands are just equation (16) and their coefficients satisfy


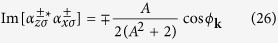



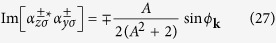


which lead to


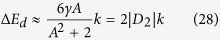


**Figure 6:**
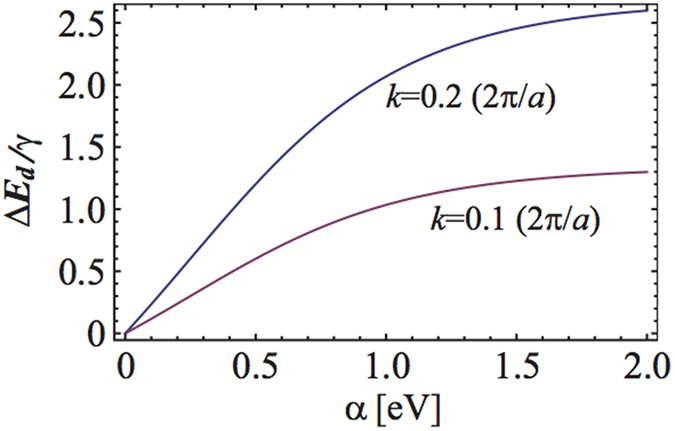
Dipole energy splitting depending on the SOC strength. Dipole energy differences as functions of the SOC for some crystal momenta.

Since this is exactly same with the energy splitting Δ*E* = *ε*_+_−*ε*_*−*_ between two bands in equation (15), this proves the origin of the band splitting near Γ point is the formation of the dipole momentum by the ISB terms. The dipole energy splitting is drawn in Fig. 6 as a function of the SOC strength. We note that Δ*E*_*d*_ saturate to a finite value Δ*E*_*d*_(*α* → *∞*) = 2*γk* in the strong SOC limit while it depends linearly on the SOC in the weak SOC region. Although we only considered the lowest bands, we arrive at the same conclusion in the same way for other ones.

In summary, first-principles and tight binding calculations on Bi and Sb triangular monolayers under electric field have been done as model systems. It is shown that the different asymmetric charge distributions are induced for each band given by Rashba splitting, and their dipole energies are quantitatively calculated under external electric field. The electric dipole energy difference and the split energy by Rashba splitting give remarkable agreement. Also, the tight binding calculation supports that the Rashba splitting originates from the electric dipole energy difference in the strong atomic spin-orbit coupling regime. We show that the electric dipole energy is mainly responsible for the Rashba band splitting.

## Methods

To investigate the surface states of Bi and Sb without bulk state contributions, we considered Bi and Sb triangular monolayers. In order to break the inversion symmetry, we applied an electric field along the direction perpendicular to the layer. For the noncollinear density functional theory (DFT) calculation, we used projector augmented-wave (PAW) method as implemented in the Vienna *ab initio* simulation package (VASP)^18^,^19^. The generalized gradient approximation (GGA) of Perdew-Burke-Ernzerhof (PBE)^20^ was used as exchange correlation functional and the spin-orbit interaction was included. The plane-wave cut off is 450 eV and the convergence criterion for energy is 10^−6^ eV difference between two sequential steps. First, the volume and internal atomic positions of bulk Bi and Sb were optimized till the internal atomic force becomes less than 10^−4^ eV/Å. Using this converged cell parameters of *a*_Bi_ = 4.641 Å and *a*_Sb_ = 4.388 Å, we constructed Bi and Sb triangular monolayers under an electric field. We then calculated their band structures, and extracted wave function characteristics as well as charge density at a given crystal momentum **k** and band index *n*. The wave function characteristics which were calculated by projecting the orbitals onto spherical harmonics of the atom were used to calculate the expectation values of the OAM at a specific **k** point. From the charge density around a Bi or Sb atom, we calculated the electric dipole moment of the orbital as


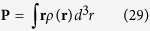


where **r** is the coordinate vector centered at the atom and *ρ*(**r**) is the charge density at **r**.

## Additional Information

**How to cite this article**: Hong, J. *et al.* Quantitative analysis on electric dipole energy in Rashba band splitting. *Sci. Rep.* **5**, 13488; doi: 10.1038/srep13488 (2015).

## References

[b1] Murakawa H. *et al.* Detection of Berry’s phase in a bulk Rashba semiconductor. Science 342, 1490–1493 (2013). 24357313 10.1126/science.1242247

[b2] Meier L. *et al.* Measurement of Rashba and Dresselhaus spin–orbit magnetic fields. Nat. Phys. 3, 650–654 (2007).

[b3] Miron I. M. *et al.* Current-driven spin torque induced by the Rashba effect in a ferromagnetic metal layer. Nat. Mater. 9, 230–234 (2010). 20062047 10.1038/nmat2613

[b4] Miron I. M. *et al.* Fast current-induced domain-wall motion controlled by the Rashba effect. Nat. Mater. 10, 419–423 (2011). 21572411 10.1038/nmat3020

[b5] Ishizaka K. *et al.* Giant Rashba-type spin splitting in bulk BiTeI. Nat. Mater. 10, 521–526 (2011). 21685900 10.1038/nmat3051

[b6] Petersen L. & Hedegård P. A simple tight-binding model of spin–orbit splitting of sp-derived surface states. Surf. Sci. 459, 49–56 (2000).

[b7] Yaji K. *et al.* Large Rashba spin splitting of a metallic surface-state band on a semiconductor surface. Nat. Commun. 1, 17 (2010). 20975678 10.1038/ncomms1016 PMC2909720

[b8] Bian G., Miller T. & Chiang T.-C. Rashba splitting and dichroism of surface states in Bi/Ag surface alloy. J. Electron Spectrosc. Relat. Phenom. 201, 36–41 (2014).

[b9] Park S. R., Kim C. H., Yu J., Han J. H. & Kim C. Orbital-angular-momentum based origin of Rashba-type surface band splitting. Phys. Rev. Lett. 107, 156803 (2011). 22107313 10.1103/PhysRevLett.107.156803

[b10] Kim B. *et al.* Microscopic mechanism for asymmetric charge distribution in Rashba-type surface states and the origin of the energy splitting scale. Phys. Rev. B 88, 205408 (2013).

[b11] Park S. R. & Kim C. Microscopic mechanism for the Rashba spin-band splitting: Perspective from formation of local orbital angular momentum. J. Electron Spectrosc. Relat. Phenom. 201, 6–17 (2015).

[b12] Kim B. *et al.* Spin and orbital angular momentum structure of Cu(111) and Au(111) surface states. Phys. Rev. B 85, 195402 (2012).

[b13] Park J.-H., Kim C. H., Rhim J.-W. & Han J. H. Orbital Rashba effect and its detection by circular dichroism angle-resolved photoemission spectroscopy. Phys. Rev. B 85, 195401 (2012).

[b14] Park S. R. *et al.* Chiral orbital-angular momentum in the surface states of Bi_2_Se_3_. Phys. Rev. Lett. 108, 046805 (2012). 22400876 10.1103/PhysRevLett.108.046805

[b15] Jung W. *et al.* Warping effects in the band and angular-momentum structures of the topological insulator Bi_2_Te_3_. Phys. Rev. B 84, 245435 (2011).

[b16] Reinert F., Nicolay G., Schmidt S., Ehm D. & Hüfner S. Direct measurements of the *L*-gap surface states on the (111) face of noble metals by photoelectron spectroscopy. Phys. Rev. B 63, 115415 (2001).

[b17] Liu Y. & Allen R. E. Electronic structure of the semimetals Bi and Sb. Phys. Rev. B 52, 1566 (1995). 10.1103/physrevb.52.1566 9981218

[b18] Kresse G. & Furthmüller J. Efficient iterative schemes for ab initio total-energy calculations using a plane-wave basis set. Phys. Rev. B 54, 11169 (1996). 10.1103/physrevb.54.11169 9984901

[b19] Kresse G. & Furthmüller J. Efficiency of ab-initio total energy calculations for metals and semiconductors using a plane-wave basis set. Comput. Mater. Sci. 6, 15–50 (1996). 10.1103/physrevb.54.11169 9984901

[b20] Perdew J. P., Burke K. & Ernzerhof M. Generalized gradient approximation made simple. Phys. Rev. Lett. 77, 3865 (1996). 10062328 10.1103/PhysRevLett.77.3865

